# Major differences in microRNA quantification are platform and sequence dependent

**DOI:** 10.1186/1753-6561-6-S6-P7

**Published:** 2012-10-01

**Authors:** Ester Feldmesser, Dena Leshkowitz, Yisrael Parmet, Shirley Horn-Saban

**Affiliations:** 1 Weizmann Institute of Science, Biological Services, Rehovot 76100, Israel; 2 Ben-Gurion University of the Negev, Industrial Engineering and Management, Beer Sheva 84105, Israel

## Background

Small RNAs (sRNAs) are known to play an important regulatory role in a wide range of organisms and biological processes through expression regulation of a diverse array of genes. Several classes of sRNA including plant microRNAs (miRNAs), small interfering RNAs (siRNAs) and Piwi-interacting RNAs (piRNAs) carry an *O*-methyl modification at their 3´ termini, and this is suspected to complicate their accurate quantification. miRNA precursors have been detected in cell lines and other tissues, where their amount does not necessarily correlate with the amount of their mature miRNA due to different regulation processes controlling their biogenesis. Methods currently used to identify and quantify sRNAs face unique challenges due to their short length, as well as the high sequence similarities between them. The correct identification, discrimination and profiling of the different types of sRNAs, including their available isoforms, are crucial for understanding of the regulatory networks they are involved in.

## Materials and methods

To compare between three high-throughput commercial platforms (Agilent and Affymetrix microarrays and Illumina next-generation sequencing (NGS)), two different approaches were applied: (a) endogenous human placenta miRNA quantification and ranking were assessed across the platforms, and (b) 12 synthetic transcripts were artificially spiked into human placenta total RNA at known input amounts and further analyzed. These transcripts included six mature miRNAs, three precursor miRNAs and three miRNAs carrying an *O*-methyl 3'modification.

## Results

Clear differences in the endogenous miRNA expression ranking were observed between the three platforms, yet Agilent and NGS showed the highest fit. When ranking miRNA expression values, none of the miRNAs were similar across all platforms. Furthermore, all the miRNAs diverged in expression ranking between at least two platforms. To understand these differences, a detailed exploration of miRNA attributes related to sequence structure and composition, including the number of observed isoforms different from the canonical miRNA, was performed. We demonstrate that the percentage of G, T, A, GG, TT, GC and TA in the sequence, RNA folding energy, number of isoforms, mismatches and 5' insertions per miRNA were significantly different between different ranking levels. For the three platforms used, the amounts of spiked-in miRNAs introduced and detected demonstrated linear correlations. However, there was a large variation in the intensity values observed for different spiked-in miRNAs at a certain concentration: up to 500 in Affymetrix, 10 in NGS, and 5 in Agilent. All platforms have a reduced capability (as low as 6%) of detecting the spiked-in *O*-methyl modified miRNAs in comparison with their matching mature miRNAs; nevertheless modified miRNAs are biologically active. In contrast to the expected, precursor miRNAs were detected, although mostly at low levels. Such signals can be erroneously interpreted as mature miRNA in the Agilent and NGS platforms.

## Conclusions

All tested platforms do not capture the absolute miRNA quantity and therefore are applicable for relative but not for absolute abundance studies. The power of miRNA detection strongly depends on the platform used and on miRNA sequence attributes and modifications.

